# Robotic vs. laparoscopic surgery at the operational level: an investigation of surgeons’ perspectives

**DOI:** 10.1007/s00464-025-12152-y

**Published:** 2025-09-02

**Authors:** Xiaodong Chen, Theresa N. Wang, Ankit Sarin, Ankit Patel, Abubaker Ali, Sarah Samreen, Jackie Cha, Dimitrios Stefanidis

**Affiliations:** 1 Department of Surgery, The Ohio State University, 395 W 12th Ave, Columbus, OH 43210 USA; 2 Davis Department of Surgery, The University of California, Sacramento, CA USA; 3 Department of Surgery, The Emory University, Atlanta, USA; 4 Department of Surgery, The Wayne State University, Detroit, USA; 5 Medical Branch Department of Surgery, The University of Texas, Galveston, USA; 6 Department of Industrial Engineering, The Clemson University, Carolina, USA; 7 Department of Surgery, The Indiana University, Indianapolis, USA

**Keywords:** Robotic-assisted surgery, Utilization performance, Surgeon survey

## Abstract

**Introduction:**

The Robotics Committee of the Society of American Gastrointestinal and Endoscopic Surgeons (SAGES) conducted a study of surgeons’ perspectives on robotic-assisted surgery (RAS) as compared to laparoscopic surgery (LS) in four domains: performance, requirements, challenges, and surgical care outcomes.

**Methods:**

An exploratory sequential mixed-methods study was performed with a thematic analysis of surgeon interviews using the framework method, followed by an online survey of SAGES Robotics Committee members. Descriptive statistics, t-tests, and ANOVA were utilized for analysis.

**Results:**

Seven robotic surgeons (3 female, 4 male) were interviewed. The primary themes were that RAS outperformed LS in (1) device performance, (2) intraoperative teaching, and (3) physical fatigue. Three perceived drawbacks of RAS compared to LS were(1) requiring more resources, (2) mechanical malfunction, and (3) care delivery cost.

55 of 92 surgeon committee members (59.8%) completed the survey. 50.9% (28/55) were male, 80% (44/55) practiced in an academic setting, and 70.9% (39/55) learned RAS during residency/fellowship training. Survey results were consistent with interview themes. Participants indicated that RAS improved performance and was associated with improved patient outcomes. They recognized the relative increased cost, the lack of tactile feedback, logistical challenges, and the increased demands of operative staff. 36.4% (20/55) surgeons ranked “AI-assisted navigation/guidance” as the “most wanted” new RAS function.

**Conclusion:**

The findings from this study provide useful insights into surgeon perspectives related to RAS as it compares with laparoscopy and desired areas for new RAS developments that may be helpful to surgical organizations and industry partners alike.

## Introduction

Although laparoscopic surgery (LS) is the dominant minimally invasive surgery approach, robotic-assisted surgeries (RAS) have experienced significant growth [1, 2] with general surgery procedures involving RAS increasing from 1.8 to 17.0% between 2012 and 2022, reflecting an annual growth rate of approximately 15%. By 2023, more than 60,000 surgeons – across specialties – around the world were performing RAS [3], leading to an increasing number of surgeons juggling between RAS and LS in their daily practice. The global RAS market, currently valued at $5.12 billion in 2024, is projected to expand to $15.52 billion by 2034. This rapid growth underscores the need to examine surgeons’ perspectives regarding operational safety, efficiency, and surgical outcomes of RAS.

While numerous studies have explored surgeons’ RAS learning curves [4–6], patient satisfaction [7], and outcome comparisons between RAS and LS [8–10], limited research has been conducted on surgeons’ operational experience and their satisfaction using RAS platforms. Understanding these experiences is critical not only for RAS device manufacturers aiming to improve device design and support but also for institutions to optimize resources and training tailored to surgeons’ needs in both RAS and LS.

To bridge this gap, the Robotics Committee of the Society of American Gastrointestinal and Endoscopic Surgeons (SAGES) conducted a mixed-methods study to gather and evaluate surgeons’ experience and perspectives on the advantages and disadvantages of utilizing RAS compared to LS. Since one measurement of robotic surgical system success is user (surgeon) satisfaction, which is influenced by the robotic system performance, usability and accessibility (i.e., how easy the robotic system is to use and/or interact with), functionality (i.e., how well the robotic system performs its intended functions), as well as vendor support quality [11, 12]. Therefore, this study focused on four operational domains: performance, device requirements, operational challenges, and surgical care outcomes from surgeons’ perspective.

## Methods

### Study design and participants

This study utilized an exploratory sequential mixed-methods design, combining qualitative and quantitative approaches to evaluate surgeon perspectives on RAS compared to LS. First, semi-structured qualitative interviews were conducted to gather surgeons’ perspectives on four operational domains: performance, device requirements, operational challenges, and surgical care outcomes. Then, findings from these interviews informed the development of a survey, which was distributed to SAGES Robotics Committee surgeon members to assess the generalizability of the interview results.

### Data collection and analysis

#### Qualitative interviews

A purposeful sampling approach was employed to recruit surgeons with experience in RAS, ensuring diversity in terms of the following criteria: gender, timing of RAS exposure (during or after residency/fellowship training), and SAGES membership status.

Interview questions focused on eliciting surgeons’ experiences with RAS and LS in the four key domains (performance, device requirements, operational challenges, and surgical care outcomes) including perceived strengths, weaknesses, and areas of future improvement for RAS technology. All individual interviews were conducted using Zoom (Zoom Video Communications, Inc, San Jose, CA), transcribed, and deidentified. Thematic analysis was conducted by the research team to analyze the transcripts following a Framework Method [13], and discrepancies were resolved via team discussion until consensus was achieved. Verbal consent was obtained from all participants prior to the interview.

#### Survey development and administration

Study consent was obtained from all survey participants. Based on the qualitative interview findings, a 17-item anonymous online survey was developed via the Qualtrics system (SAP SE, Provo, UT). The survey included six demographic questions, ten 4-point Likert-scale questions comparing RAS and LAS experience across the four domains, and one open-ended question for additional feedback and recommendations. The online survey was distributed to all SAGES Robotics Committee surgeon members via email.

#### Quantitative data analysis

Quantitative results were used to determine the generalizability of the interview themes across a broader population. Descriptive statistics, t-tests, and ANOVA were performed to analyze the demographic questions and Likert-scale questions in the survey.

## Results

### Qualitative interviews

Seven surgeons (three female, four male) with RAS experience participated in 30-min, semi-structured interviews conducted between January and February 2024. The majority (71.4%, 5/7) had learned how to operate on a RAS platform during residency or fellowship training. Two of the interviewed surgeons (28.6%) were not SAGES members.

Based on the themes highlighted below, surgeons felt RAS outperformed LS in three operational areas:

(1) Device Performance—Surgeons perceived that RAS outperformed LS in accuracy, precision, accessibility, and movement. However, the lack of tactile feedback was a notable limitation. As one surgeon remarked:

“For accuracy and precision, I think the robot is better (than laparoscopy)… having the articulated approach does allow you to get into a little bit more precise places…I do think (lack of tactile feedback) that’s a concern with the robot, but I think it’s one that you can overcome by using visual cues to understand tension and tissue motion…” (interviewee FS1)

(2) Intraoperative Teaching—The dual console of RAS systems facilitated easier teaching and skill transfer compared to LS. One participant stated:

“I think the robot is definitely better for (intraoperative) teaching (than laparoscopy). You’re just able to pass it back and forth (between a resident) a lot easier when you have a (robotic surgical system with) dual console.” (interviewee FS2)

(3) Physical and/or Visual Fatigue—RAS was associated with reduced physical and/or visual fatigue compared to LS. For example, one surgeon explained:

“If I did a difficult laparoscopic lithotomy, I would sit between the (patient) legs. I’d be doing this: looking up, trying to sew, straight sticks…If I did it with robot, I would be sitting at the console, much less fatiguing for me… I still get tired just because of the mental strain for the other cases, but I’m much less (physical) fatiguing.” (interviewee MS1)

However, when compared to LS, surgeons also noted three main drawbacks of RAS:

(1) Resource Intensive—RAS usually required a larger operating room (OR) and a proficient team who was familiar with RAS in order to set up the OR and progress through the case efficiently. Additionally, given the cost of the robotic system, most hospitals have not yet been able to install a robotic system in each OR, leading to difficulty in scheduling RAS cases. Furthermore, insurance reimbursement also sometimes contributed to barriers to RAS case scheduling. One surgeon noted:

“The (operating) room is a problem… you need a (operating) room, you need the robot, and you need staff who can take care of that… because the robot is only in a certain room, I have to request it well in advance to find open time… It can be pretty challenging and cumbersome to get access to the robot in this setting.” (interviewee, MS2)

(2) Mechanical Malfunction—Compared to LS, concern about mechanical malfunction of the robotic platform intraoperatively was raised. Surgeons felt that they did not have sufficient knowledge and experience to troubleshoot mechanical dysfunctions, leading to a disruption of the RAS operative workflow (e.g., conversion of RAS to LS or Open). As one surgeon commented:

“The problem with the robotic malfunction is I don’t know what’s going wrong. If a stapler or an instrument fails (in laparoscopy), I know how to fix that… (in terms of robotic malfunction) I just reset the robot and cross my fingers and hope (it would be fixed) ... The biggest issue is I don’t know what’s gone wrong (with the robot). So, if we still cannot fix it, just need to convert it to an open (case).” (interviewee MS3)

(3) Higher Care Delivery Cost—This included cost of usage as well as reimbursement. For example, a laparoscopic colectomy and a robotic colectomy use the same Current Procedural Terminology (CPT) code for billing and reimbursement, but the total costs of performing a robotic colectomy were higher than a laparoscopic colectomy. It was suggested that the surgeons’ experience and knowledge of RAS disposable costs may help to increase cost-efficiency. As one surgeon said:

“I try to be conscientious of what instruments I’m using (in the RAS case) and have knowledge of what the cost of those instruments are. Because if it’s not put in the robot and activated, it doesn’t get charged for that case and I think surgeons should know that.” (interviewee FS1)

### Quantitative survey

**Fig. 1:**
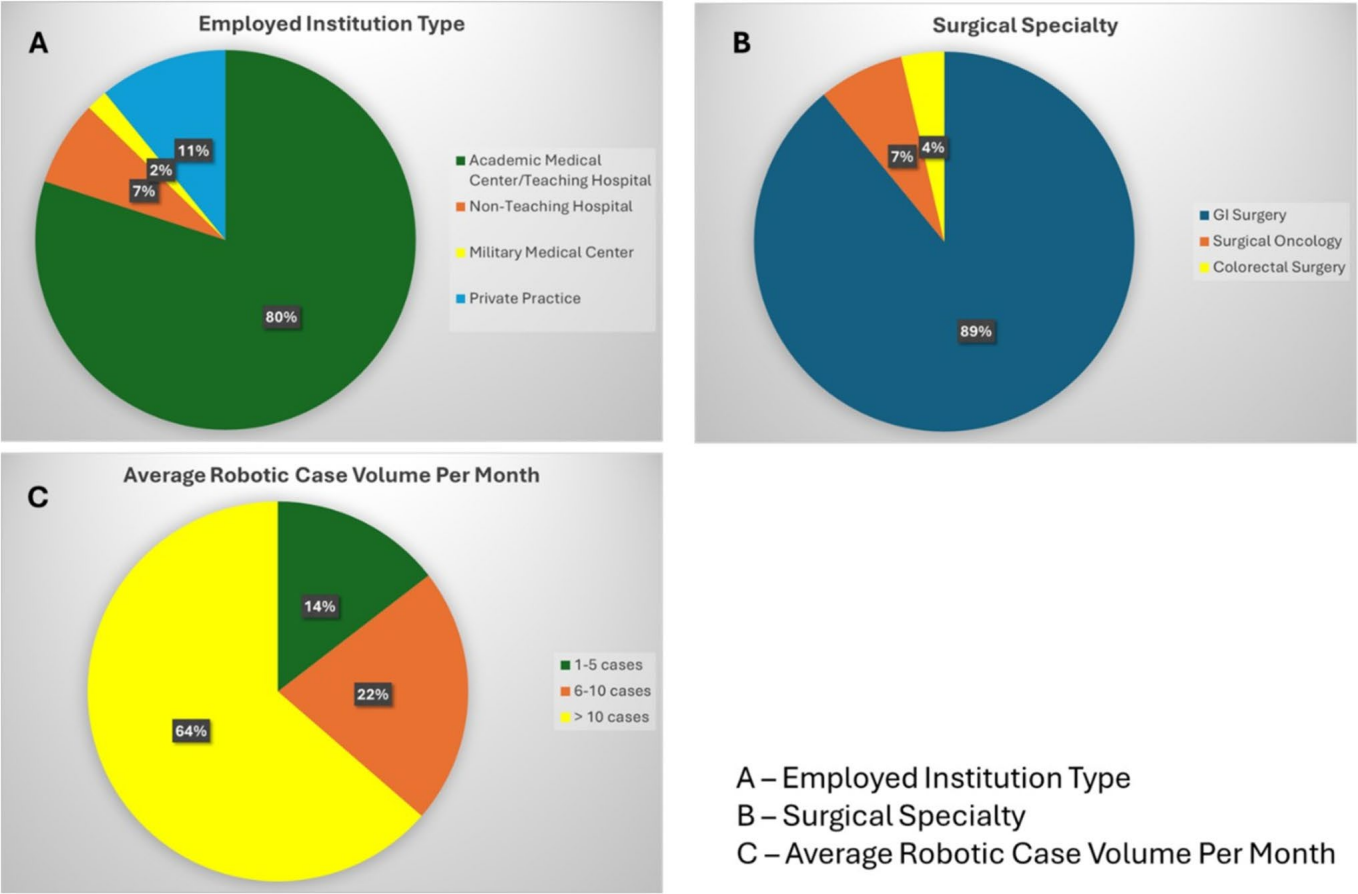
Participant information

A total of 55 SAGES Robotics Committee surgeons (59.8% response rate) completed the survey (Fig. 1). Respondents were male (50.9%, 28/55), practiced in academic settings (80%, 44/55), and had learned RAS during residency or fellowship (70.9%, 39/55). Most participants (63.6%, 35/55) performed over 10 RAS cases per month, with 5.5% (3/55) also having experience using systems other than the da Vinci platform (Intuitive Surgical, Sunnyvale, CA, USA).

Survey results confirmed interview themes and revealed additional insights (Table 1).

**Table 1:** Comparison between robotic and laparoscopic surgery

Metrics*	RAS Mean (± SD)	LS Mean (± SD)	p
Utilization—Performance Domain (Score 4 = Best Performance)			
Accuracy and precision	3.80 (0.62)	2.45 (0.63)	< 0.001
Access to difficult areas	3.84 (0.63)	2.36 (0.80)	< 0.001
Latency in movement	3.49 (0.72)	3.29 (0.99)	0.229
Facilitation of OR teaching	3.56 (0.74)	2.57 (0.81)	< 0.001
Tactile feedback	1.60 (0.97)	3.31 (0.84)	< 0.001
Frequency of mechanical malfunctions	2.78 (0.94)	2.96 (0.79)	0.274
Utilization—Requirement Domain (Score 4 = Least Requirements)			
Operating room care team support	2.29 (1.13)	2.65 (0.89)	0.064
Operating room scheduling	2.27 (1.10)	3.20 (1.01)	< 0.001
Operating room space requirements	2.02 (0.95)	2.95 (0.89)	< 0.001
Malfunctions troubleshooting tech support	2.51 (1.00)	2.60 (0.81)	0.600
Utilization—Challenge Domain (Score 4 = Totally Not a Challenge)			
Learning curve of the technique	3.31 (0.74)	2.07(0.84)	< 0.001
Surgeon physical strain/ fatigue	3.38 (0.87)	1.75 (0.80)	< 0.001
Surgeon visual strain/ fatigue	3.56 (0.69)	2.15 (0.78)	< 0.001
Grant trainee autonomy	3.15 (0.80)	2.29 (0.76)	< 0.001
DIY troubleshooting malfunctions	2.55 (1.00)	2.78 (0.85)	0.185
Utilization—Care Outcome Domain (Score 4 = Best Performance)			
Length of procedure	2.89 (0.96)	2.76 (0.61)	0.407
Blood loss	3.67 (0.58)	3.09 (0.75)	< 0.001
Wound infection	3.75 (0.62)	3.53 (0.74)	0.096
30-day readmission	3.71 (0.60)	3.49 (0.74)	0.093
Intraoperative complications	3.65 (0.55)	3.18 (0.72)	0.0002
Total patient care delivery cost	2.22 (1.12)	3.11 (0.74)	< 0.001

^*^All used a 4-point Likert rating scale with higher scores indicative of better performance

In the “utilization performance” domain, surgeon participants indicated that when compared to LS, RAS significantly improved the operational performance in three out of six aspects: “Access to Difficult Areas” (3.84 > 2.36, *p* < 0.001), “Accuracy and Precision” (3.80 > 2.45, *p* < 0.001), and “Facilitation of OR Teaching” (3.56 > 2.57, *p* < 0.001). However, RAS significantly underperformed LS in “Tactile Feedback” (1.60 < 3.31, *p* < 0.001). In general, “Frequency of Mechanical Malfunctions” was reported to be similar between RAS and LS. Female surgeons demonstrated lower satisfaction with the frequency of mechanical malfunctions in RAS than male surgeons (2.48 < 3.07, *p* = 0.018).

In the “utilization requirements” domain, compared to RAS, LS cases were noted to be significantly easier to schedule (3.20 > 2.27, *p* < 0.001) and more OR-space-friendly (2.95 > 2.02, *p* < 0.001). On average, surgeons perceived that “Malfunction troubleshooting tech support” was similar between RAS and LS, but female surgeons indicated insufficient technical support for troubleshooting robotic surgical system mechanical malfunctions specifically (2.19 < 2.82, *p* = 0.017).

In the “utilization challenges” domain, surgeons reported perceiving an easier learning curve for RAS than LS (3.31 > 2.07, *p* < 0.001). Whether a surgeon had learned RAS during residency or fellowship training had no impact on their perceived RAS learning curve score (3.31 vs. 3.31). Surgeons also reported feeling that it was significantly easier and more convenient to grant a trainee autonomy in RAS than in LS. Compared to LS, surgeons indicated Do-It-Yourself (DIY) troubleshooting mechanical malfunctions in RAS was more difficult.

In the “utilization care outcomes” domain, surgeons perceived that RAS significantly outperformed LS in two aspects: “Intraoperative Complications” (3.65 > 3.18, *p* = 0.0002) and “Blood Loss” (3.67 > 3.09, *p* < 0.001). LS had a perceived superior performance in “Total Patient Care Delivery Cost” (3.11 > 2.22, *p* < 0.001) compared to RAS. In terms of the “Length of Procedure,” male surgeons perceived RAS as having better time-efficiency outcomes than female surgeons (3.18 > 2.59, *p* = 0.022), but reported similar scores for LS (2.75 vs 2.78).

We also asked surgeon participants to rank their desired extra RAS functions that could be added on in future, as well as to grade the helpfulness of support needed from and provided by SAGES (Table 2). Among the four options, 36.4% (20/55) surgeons ranked “Artificial Intelligence (AI)-assisted Navigation/Guidance” as the “most wanted” new RAS add-on function while 30.9% (17/55) selected it as the “least wanted” new RAS function. The second “most wanted” new RAS function was “Flexible Ergonomic Design” (30.9%, 17/55), followed by “Error Movement/Malfunction Warning System” (21.8%, 12/55). Regarding the support needed from SAGES, the three highest ranked support items were as follows: “Develop Robotic Surgery Skills Curricula for Trainees and Practicing Surgeons” (3.56 ± 0.57), “Develop Best Practice Guidelines for Robotic Surgery” (3.44 ± 0.81), and “Develop Tools for Robotic Surgery Performance Assessment” (3.42 ± 0.76).

**Table 2:** List of desired additional robotic functions and SAGES Help/support

Desired Additional Future Robotic Functions Ranking Options	
• AI-assisted navigation/ guidance	
• Error movement/malfunction warming system	
• Flexible ergonomic design	
• Tele-team communication	
SAGES Help/Support Needed (Score 4 = Very Helpful)	
• Develop white paper outlining RAS troubleshoot steps at operational level	
• Develop robotic surgery skills curricula for trainees and practicing surgeons	
• Develop assessment tools for robotic surgery systems	
• Develop tools for robotic surgery performance assessment	
• Develop certification programs for robotic surgeon skill similar to FLS and FES	
• Develop a registry for existing and new robotic systems data deposit	
• Develop best practice guidelines for robotic surgery	
• Delphi consensus on essential steps of core RAS procedures	
• Develop training module for mechanical malfunction prevention and DIY troubleshooting	

## Discussion

RAS is increasingly recognized as a key component of minimally invasive surgery, with the potential to become a mainstream approach for routine operations in the near future [14]. Surgeons are the primary operators of RAS; their operational experience and perspective are vital for improvements in robotic surgical devices, patient care delivery, and surgeon wellbeing. Our study first interviewed surgeons and identified three RAS strengths (device performance, intraoperative teaching, and physical fatigue) and three weaknesses (requiring more resources, mechanical malfunctions disrupting operative workflow, and higher care delivery costs) at the operational level in comparison with LS. We subsequently developed a questionnaire based on the interview findings and surveyed SAGES Robotic Committee members. The survey results suggest that our qualitative interview findings can be generalized to a broader RAS surgeon population.

Overall, participating surgeons agree that the RAS device utilization performance and care outcomes are better than LS in most surveyed aspects (Table 1), which are consistent with current literature [15–17]. However, the lack of tactile or haptic feedback to the surgeon and higher care delivery costs are perceived as two operational challenges. In LS, tactile (haptic) feedback enables surgeons to feel the touch/force/resistance when manipulating a surgical instrument, aiding surgeons to be more accurate and dexterous at operating in the OR [18]. Currently, most robotic surgical systems are not equipped with haptic feedback, which may make it harder for surgeons and surgery residents to transit between LS and RAS in daily practice, potentially leading to decreased efficiency and increased cost of care delivery in RAS [19]. The concern regarding haptic feedback is that the lack of this feedback may result in accidental manipulation of a robotic instrument with excessive force or manipulation of tissue unintentionally. The risk of this may be higher with surgical trainees. One surgeon commented in the interview that “I see that (controlling a wrong instrument poking somewhere accidently) a lot in my trainees and that can be really dangerous.” Therefore, providing training to enhance surgeons and residents’ recognition and knowledge of RAS visual cues of tension/force may be helpful to overcome these challenges. Fortunately, newer robotic systems are now incorporating haptic feedback that may address this limitation [20].

Mechanical malfunction is another challenge concerning surgeons when they perform RAS. Based on surgeon experiences, RAS mechanical malfunctions may include the robotic surgical system or instrument unexpectedly freezing, robotic arms bumping together or with something else, or other various faults. Unless a surgeon has sufficient knowledge and skills to troubleshoot mechanical malfunctions in RAS, the operative workflow is at risk of being disrupted, bringing uncertainty into care outcomes as well as mental pressure and anxiety to the operating surgeon and care team in the OR. For example, one surgeon recalled that “We had a critical fault with the robot…in the middle of the operation, we had to literally restart the entire Da Vinci system while the instruments were in the patient. That’s scary. Another thing that’s scary is when you can’t withdraw an instrument for one reason or another, when it’s stuck on tissue, or won’t open and close… you have to use extra devices to try and break it through.” The study results also found that female surgeons, more than male surgeons, felt that there was insufficient technical support for RAS mechanical malfunctions. We recommend that surgical robotic device manufacturers further enhance their step-by-step troubleshooting instructions for surgeons and the surgical team.

In addition, our survey findings reveal that our committee members perceive that a robotic surgery skills curriculum developed by SAGES would be most helpful for trainees and practicing surgeons. Although numerous studies have been conducted to develop RAS training curricula for trainees and young surgeons [21–24], a standard robotic skills training curriculum from a surgical association like SAGES would offer a reliable skill development framework to help surgeons and residents regulate and improve RAS performance systematically. Our survey results also reveal an interesting bipolar trend concerning the desirability of integrating AI-assisted navigation into the robotic surgical system – 36.4% voted this as a “most wanted” feature while 30.9% voted it as a “least wanted” feature. This may suggest that there is both excitement and anxiety about applying AI to RAS. One potential reason may be that RAS surgeons not only are optimistic about possible AI advantages (e.g., decision-making support), but also concern about potential risks (e.g., data breaches) exacerbated by AI applications [25]. Further exploration is needed to investigate this topic.

This study has several limitations. The small interview sample size (*N* = 7) was sufficient to achieve thematic saturation but limits the generalizability of the qualitative findings. Additionally, the survey response rate (59.8%) may introduce response bias, potentially skewing the representativeness of the results. Since all interviewees had experience with the Da Vinci platform as their primary robotic tool, these results may be more platform-specific and not as generalizable to other future platforms. Future studies should aim for larger and more diverse samples to validate and expand on these findings.

In conclusion, this study provides valuable insights into the operational strengths and weaknesses of RAS as perceived by surgeons. These findings have important implications for device manufacturers, surgical societies, and hospitals aiming to optimize RAS systems, training, and support resources. Continued efforts to address identified challenges, such as tactile feedback limitations and mechanical malfunctions, will further enhance the integration of RAS into routine surgical practice.

## Data availability statement

The data that support the findings of this study are available from the corresponding author upon reasonable request. Data are available from the corresponding authors with the permission of the Society of American Gastrointestinal and Endoscopic Surgeons (SAGES).
